# Resternotomy does not adversely affect outcome after left ventricular assist device implantation

**DOI:** 10.1186/s40001-017-0289-2

**Published:** 2017-11-15

**Authors:** Maria Papathanasiou, Loukas Tsourelis, Nikolaus Pizanis, Achim Koch, Markus Kamler, Tienush Rassaf, Peter Luedike

**Affiliations:** 1 Department of Cardiology and Vascular Medicine, West German Heart and Vascular Center, University Hospital Essen, Hufelandstr. 55, 45147 Essen, Germany; 2 Department of Thoracic and Cardiovascular Surgery, West German Heart and Vascular Center, University Hospital Essen, Hufelandstr. 55, 45147 Essen, Germany

**Keywords:** Resternotomy, Left ventricular assist device, Redo-surgery

## Abstract

**Background:**

Resternotomy in cardiac surgery is considered a risk factor for postoperative complications. Previous studies have demonstrated an ambiguous relationship between resternotomy and clinical outcomes. Registry data from a mixed population of durable circulatory support devices suggest that history of cardiac surgery is a risk factor for mortality. Our study investigates the prognostic significance of resternotomy in a homogenous cohort of left ventricular assist device (LVAD) recipients.

**Methods:**

The study included adult patients receiving a continuous-flow LVAD at our institution during the period 2010–2016. Postoperative adverse events and length of stay were analyzed. Survival was assessed at 6 months and by the end of the study. Multivariate risk factor analysis was conducted for independent predictors of death.

**Results:**

One hundred twelve patients, who received an intrapericardial LVAD (HVAD, HeartWare), were included in our analysis. Twenty-four patients (21.4%) had a history of previous sternotomy. These patients were older and non-eligible for bridging, and had more frequently coronary heart disease. Univariate analysis demonstrated no differences in the observed complications postoperatively. Survival was similar among groups. Destination therapy was the only predictor of mortality in our analysis (*p* = 0.02).

**Conclusions:**

Resternotomy was not associated with worse outcomes after LVAD implantation in our cohort.

## Background

Left ventricular assist device (LVAD) therapy has revolutionized the management of patients with advanced heart failure. Following device approval for destination therapy (DT), LVAD implantations have dramatically increased with nearly half of all implants currently assigned to DT [1, 2]. Accordingly, a right shift in patient age, burden of comorbidities, and functional status was noticed with a significant proportion of patients having previously undergone median sternotomy for cardiac surgery. Although device technology and operative techniques continue to improve and the future of LVAD therapy is believed to be minimally invasive, the current standard approach for device implantation involves median sternotomy with cannulation of the right atrium and ascending aorta for institution of extracorporeal circulation. Resternotomy is considered a surgical challenge, as it is traditionally associated with an increased risk for perioperative complications, including excessive bleeding, sternal wound infection, right ventricular dysfunction, and injury of cardiac structures and coronary grafts [3].

Previous studies investigating the prognostic significance of resternotomy in non-LVAD procedures have yielded controversial results [3–11]. The largest registry of durable mechanical circulatory support (MCS) devices reported an association between previous cardiac surgery and mortality risk in patients receiving LVAD or biventricular support with a wide range of available devices [1, 2]. Our study aimed to evaluate the impact of previous sternotomy on postoperative outcome and survival in a homogenous cohort consisting exclusively of intrapericardial, continuous-flow LVAD recipients.

## Methods

### Study design

We retrospectively reviewed the database of our interdisciplinary heart failure unit to identify consecutive adult patients who received a continuous-flow LVAD at our institution from December 2010 through June 2016. Clinical data regarding patients’ medical history and disease status in the recent preoperative period, as well as operative variables, were prospectively collected in a digitalized database dedicated to clinical surveys. The Interagency Registry for Mechanically Assisted Circulatory Support (INTERMACS) classification for advanced heart failure was used to describe the preoperative clinical status of the study patients. Accordingly, patients were stratified to one of seven INTERMACS profiles: Profile 1: critical cardiogenic shock, Profile 2: progressive decline despite inotropic therapy, Profile 3: stable but inotrope dependent, Profile 4: resting symptoms, Profile 5: exertion intolerant, Profile 6: exertion limited, and Profile 7: Advanced New York Heart Association Class III. Postoperative complications and follow-up data were extracted retrospectively from the surgical reports and the patients’ electronic health records. The follow-up visits in the outpatient clinic were prospectively scheduled at 1, 2, 3, and 6 months after implantation according to an established internal protocol. Additional visits or inpatient treatments were planed depending on the clinical course and the occurrence of adverse events. The study received Institutional Review Board approval.

### LVAD implantation procedure

The continuous-flow HVAD (HeartWare International Inc., Framingham, MA) was the implanted LVAD at our institution since 2010. All implantations were performed by the same surgical team. The procedure included median sternotomy and cannulation of the right atrium and the ascending aorta for institution of normothermic cardiopulmonary bypass, which is the most widely adopted surgical technique to date.

### Outcome measures

Postoperative recovery was evaluated by univariate analysis of the following variables: need for temporary right ventricular assist device (RVAD) not planned preoperatively, re-exploration for refractory intrathoracic bleeding or pericardial tamponade, duration of invasive ventilation, postoperative tracheostomy, duration of ICU and hospital stay. Hospitalization rates and duration of hospitalization after discharge and up to 6 months, as well as infection and sternal wound infection rates, were the mid-term outcomes of the study. Secondarily, our analysis included survival estimation at 6 months and by the end of the study.

### Statistical analysis

Continuous variables are summarized as means (standard deviations) unless indicated otherwise and categorical variables as counts (percentages). Continuous data were evaluated for normality of distribution using the Shapiro–Wilk’s test. The two-sided *t* test was used for comparison of continuous, normally distributed data, otherwise the non-parametric Mann–Whitney *U* test. The Chi-square test and Fisher’s exact test were used for testing association between two categorical variables. Kaplan–Meier analysis was conducted to estimate survival for the different groups of patients. The log-rank test was performed to determine differences in survival distribution between groups. Risk factors for death were assessed by Cox proportional hazards regression analysis with a forward variable selection procedure. All variables with a significance < 0.10 were introduced in the model. Potentially relevant variables were selected based on previous reports on independent predictors of death in LVAD patients. These were resternotomy, age, male gender, left ventricular ejection fraction, DT, coronary heart disease, hypertension, diabetes, extracorporeal life support preoperatively, and invasive ventilation preoperatively. Patients were censored at the time of transplant, explantation for recovery, or by the end of the study [12]. The level of significance was set to 0.05. All analyses were performed using SPSS (IBM Corp., SPSS Statistics, Version 23.0. Armonk, NY).

## Results

Our study enrolled 112 consecutive adult patients, who received equivalent number of continuous-flow LVADs at our institution from 12/2010 through 06/2016. The majority were males (81.3%) and the mean age was 58.4 ± 10.9 years. Coronary heart disease (CHD) was the dominant etiology for heart failure (53.6%), followed by dilated cardiomyopathy (DCM) (43.7%). DT was the therapeutic strategy in 69% of implants. Approximately one-third of the study population were ambulatory heart failure patients (INTERMACS level ≥ 4). The remaining comprised inotrope-dependent, hospitalized individuals with an increased proportion of patients in critical cardiogenic shock (28% INTERMACS level 1). A total of 24 patients (21.4%) had a history of previous sternotomy for valvular heart surgery or coronary artery bypass grafting (CABG). None of the study patients had a history of previous LVAD support.

Baseline clinical characteristics for the resternotomy vs. primary sternotomy groups are summarized in Table 1. Patients with resternotomy were significantly older (mean age 61.4 vs. 57.5, *p* = 0.02). There was equal representation of males across the two groups. Ischemic etiology of heart failure was identified in the vast majority of patients in the resternotomy group and in nearly half of the patients with primary sternotomy (91.7 vs. 43.2%, *p* < 0.001). DCM was more common in the primary sternotomy group (8.3 vs. 53.4%, *p* < 0.001). Regarding the therapeutic indications, patients with previous sternotomy were less frequently bridged to transplantation/candidacy (4.2 vs. 33%, *p* = 0.005), as opposed to DT (95.8 vs. 61.4%, *p* = 0.001). All patients bridged to recovery had no history of previous cardiac surgery. Most of the patients who had a prior sternotomy were ambulatory heart failure patients in INTERMACS level 4. Conversely, in the group of primary sternotomy an even distribution to levels 1–4 was noted.

**Table 1:** Baseline characteristics of the study population

Variables	Overall (*n* = 112)	Resternotomy (*n* = 24)	Primary sternotomy (*n* = 88)	*p* value
Patient data				
Age (year)	58.4 (10.9)	61.4 (6.5)	57.5 (11.8)	*0.02*
Male gender	91 (81.3)	22 (91.7)	69 (78.4)	0.14
BMI (kg/m^2^)	26.2 (4.3)	26.4 (3.9)	26.2 (4.4)	0.54
BSA (m^2^)	1.96 (0.2)	1.95 (0.2)	1.96 (0.2)	0.93
Clinical variables				
CHD	60 (53.6)	22 (91.7)	38 (43.2)	*<* *0.001*
DCM	49 (43.7)	2 (8.3)	47 (53.4)	*<* *0.001*
Myocarditis	3 (2.7)	0 (0.0)	3 (3.4)	1.00
BTT/BTC	30 (26.8)	1 (4.2)	29 (33.0)	*0.005*
DT	77 (68.7)	23 (95.8)	54 (61.3)	*0.001*
BTR	5 (4.5)	0 (0.0)	5 (5.7)	0.85
INTERMACS 1	31 (27.7)	4 (16.7)	27 (30.7)	0.17
INTERMACS 2	18 (16.1)	4 (16.7)	14 (15.9)	1.00
INTERMACS 3	25 (22.3)	3 (12.5)	22 (25.0)	0.19
INTERMACS 4	35 (31.3)	13 (54.2)	22 (25.0)	*0.006*
INTERMACS 5	3 (2.7)	0 (0.0)	3 (3.4)	1.00
LVEF (%)	17.3 (6.6)	18.8 (6.6)	16.9 (6.5)	0.22
CI (l/min/m^2^)	1.8 (0.4)	1.9 (0.5)	1.8 (0.4)	0.43
MeanPAP (mmHg)	33.8 (10.8)	39.9 (11.3)	32.1 (10.2)	*0.03*
Comorbidities				
Arterial hypertension	86 (76.8)	20 (80.3)	66 (75.0)	0.59
CKD	54 (48.2)	12 (50.0)	42 (47.7)	0.84
Diabetes	38 (33.9)	9 (37.5)	29 (33.0)	0.68
COPD	34 (30.4)	9 (37.5)	25 (28.4)	0.39
PAD	12 (10.7)	8 (33.3)	4 (4.5)	*<* *0.001*
Preoperative interventions				
IABP	11 (9.8)	1 (4.2)	10 (11.4)	0.45
ECLS	21 (18.8)	4 (16.7)	17 (19.3)	1.00
Hemodialysis	25 (22.3)	4 (16.7)	21 (23.9)	0.45
Invasive ventilation	21 (18.8)	3 (12.5)	18 (20.5)	0.56
Surgical complexity				
CPB time (min)	94.9 (37.2)	99.3 (28.4)	93.7 (39.2)	0.49
Concomitant procedure	20 (17.9)	2 (8.3)	18 (20.5)	0.24

*BMI* body mass index, *BSA* body surface area, *CHD* coronary heart disease, *DCM* dilated cardiomyopathy, *BTT* bridge to transplant, *BTC* bridge to candidacy, *DT* destination therapy, *BTR* bridge to recovery, *INTERMACS* Interagency Registry for Mechanically Assisted Circulatory Support, *LVEF* left ventricular ejection fraction, *CI* cardiac index, *PAP* pulmonary artery pressure, *CKD* chronic kidney disease, *COPD* chronic obstructive pulmonary disease, *PAD* peripheral arterial disease, *IABP* intra-aortic balloon pump, *ECLS* extracorporeal life support, *CPB* cardiopulmonary bypass

Hemodynamic variables of left ventricular ejection fraction (LVEF) and cardiac index (CI) did not differ significantly between groups, but a higher mean pulmonary artery pressure (mean PAP) was found in the resternotomy group (*p* = 0.03). Hypertension, chronic kidney disease (CKD), chronic obstructive pulmonary disease (COPD), diabetes, and peripheral arterial disease (PAD) were the major comorbidities of interest in our study. With the exception of PAD, which was exceptionally more prevalent in the resternotomy group (33.3 vs. 4.5%, *p* < 0.001), there were no differences in comorbidities across the two groups. Furthermore, no significant differences were observed in the rates of preoperative interventions, i.e., circulatory support (ECLS, IABP), invasive ventilation, and renal replacement therapy. Variables associated with surgical complexity, i.e., cardiopulmonary bypass (CPB) time and concomitant cardiac procedures, were also similar.

**Fig. 1:**
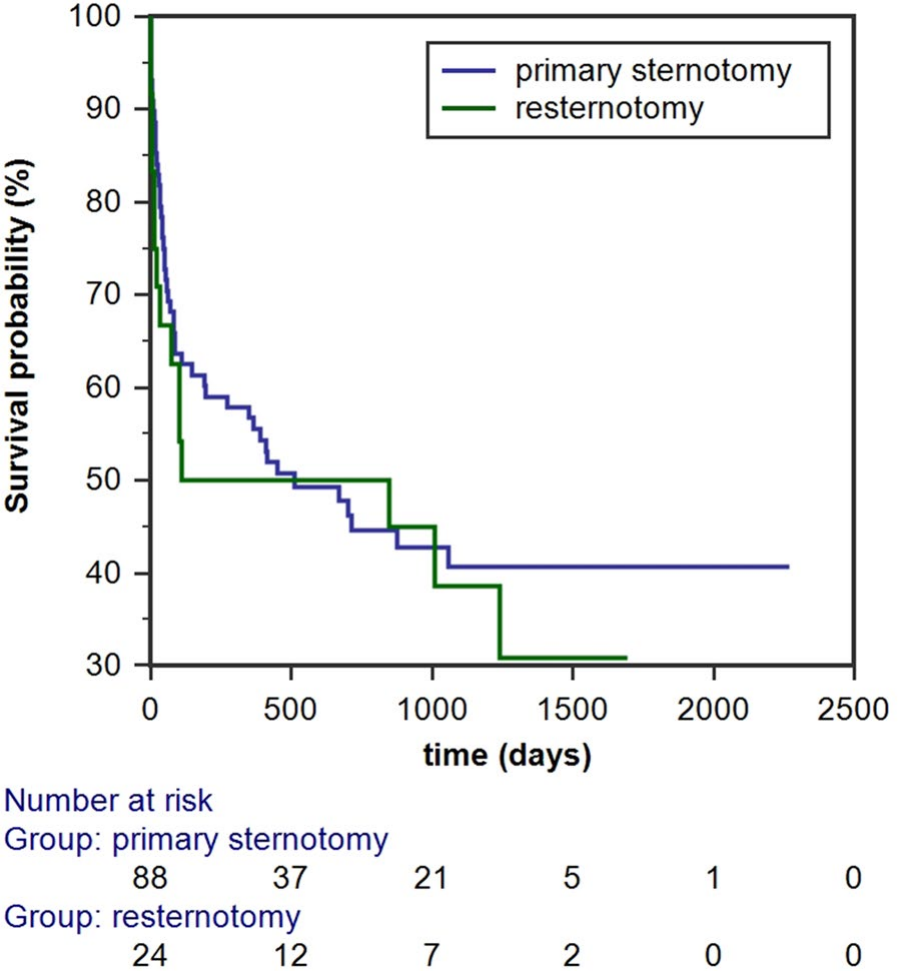
Kaplan–Meier survival analysis for patients receiving LVAD stratified by the history of previous sternotomy. Log-rank test: 0.59

The median follow-up duration for the overall population was 1188 days (min, max: 118, 2379). Analysis of postoperative adverse events did not reveal any significant differences in the rates of complications and duration of ICU and cumulative hospital stay. The observed rates of infections in general, as well as sternal wound infections, the hospitalization rates, and duration of hospitalization were similar up to 6 months post-implantation (Table 2). As depicted in Fig. 1, the Kaplan–Meier analysis did not infer a significant difference in survival probability among the two groups (Log-rank test = 0.59). The observed survival rate at 6 months post-implantation was 50% for the resternotomy group and 60.2% for the primary sternotomy group (*p* = 0.37). Cox proportional hazards regression, adjusting for multiple covariates, revealed that DT was a strong independent predictor of death with a nearly threefold increase in mortality risk (HR 2.83, *p* = 0.01) (Table 3).

**Table 2:** Comparison of postoperative and 6-month outcome

	Overall (*n* = 112)	Resternotomy (*n* = 24)	Primary sternotomy (*n* = 88)	*p* value
Postoperative				
RVAD	6 (5.4)	1 (4.2)	5 (5.7)	1.00
Re-exploration	23 (20.5)	6 (25.0)	17 (19.3)	0.57
Duration of invasive ventilation (h)	362.13 (619.6)	403.29 (536.8)	350.91 (642.7)	0.68
Tracheostomy	36 (32.1)	9 (37.5)	27 (30.7)	0.53
ICU stay (day)	18.9 (27.7)	17.3 (23.7)	19.4 (28.8)	0.72
Hospital stay (day)	43.7 (35.0)	45.2 (37.3)	43.3 (34.7)	0.82
6-Month				
Survival	65 (58.0)	12 (50.0)	53 (60.2)	0.37
Hospitalizations	0.9 (1.2)	0.5 (0.9)	1.0 (1.2)	0.11
Hospital stay (day)	12.9 (27.6)	4.3 (8.4)	14.8 (29.9)	0.24
Sternal wound infection	3 (4.6)	1 (8.3)	2 (3.8)	0.46
Overall infection	14 (21.5)	1 (8.3)	13 (24.5)	0.34

*RVAD* right ventricular assist device, *ICU* intensive care unit

**Table 3:** Cox proportional hazards regression model for mortality risk assessment

Variables	HR (95% CI)	*p* value
Resternotomy	0.86 (0.434–1.690)	0.65
Age	0.98 (0.952–1.019)	0.38
Male gender	0.84 (0.409–1.719)	0.63
LVEF	1.02 (0.977–1.061)	0.39
DT	2.83 (1.207–6.649)	*0.02*
CHD	1.06 (0.552–2.051)	0.85
Hypertension	0.88 (0.447–1.714)	0.70
Diabetes	1.32 (0.737–2.362)	0.35
ECLS	1.21 (0.506–2.880)	0.67
Invasive ventilation	1.47 (0.643–3.340)	0.36

*LVEF* left ventricular ejection fraction, *DT* destination therapy, *CHD* coronary heart disease, *ECLS* extracorporeal life support, *HR* hazard ratio, *CI* confidence interval

## Discussion

### Preoperative patients’ profile

Our analysis revealed that patients referred to LVAD implantation after previous cardiac surgery have a very complex and prognostically unfavorable clinical profile. The vast majority of patients are considered for DT, mainly in light of their more advanced age and burden of comorbidities. In this context, postoperative complications of LVAD implantation could be expected to be more pronounced in patients previously submitted to cardiac surgery via median sternotomy. For these patients, referral to implantation takes place most frequently at a fairly advanced stage of heart failure (INTERMACS profile 4), while there is a similar representation of the more critical stages in both groups (INTERMACS profiles 1, 2, and 3).

### Early postoperative outcome

The complexity of resternotomy is attributed mainly to the challenges of retrosternal adhesiolysis and preservation of sternal robustness. In particular, the associated risk of damage to cardiac structures during retrosternal tissue dissection, such as the free right ventricular wall and any patent coronary drafts, may potentially result in higher rates of major intrathoracic bleeding and severe right ventricular failure. Furthermore, sternal stability is of paramount importance for an uncomplicated wound healing. Consequently, the acute impact of resternotomy on adverse outcomes is anticipated within the early postoperative period. In our study sample, despite the higher risk profile of the patients, resternotomy was not associated with higher rates of severe bleeding, RVAD implantation, longer ventilation, and hospitalization postoperatively.

These results suggest a benign effect of the surgical complexities that are linked to resternotomy. Importantly, surgical experience and perioperative treatment algorithms have the potential to counterbalance any additive risks. Regarding our practice, standardization of perioperative management and some modifications in the redo cases may have contributed to risk elimination and better outcomes. In our center, diagnostic evaluation prior implantation included for all patients laboratory work-up with special attention to platelets and coagulation system (including platelet reactivity and von Willebrand factor), ECG, chest X-ray, echocardiogram, coronary angiography, right heart catheterization, and in most cases cardiopulmonary exercise testing. An additional chest CT scan was performed in most of the redo cases, to localize graft position and position of the heart relative to the sternum. Low-dose aspirin was given continuously in both groups before and after surgery. Any other antiplatelet therapies, such as ticagrelor, were discontinued for 48 h, while anticoagulants were switched to intravenous unfractionated heparin. Perioperative antibiotic prophylaxis (1 h prior incision, 8 and 16 h post-incision) was also similar. To open the sternum instead of a saber saw, an oscillating saw was implemented in redo cases. In the latter, the first sternal lamella was divided, ventilation was stopped, and thereafter, under moderate sternal elevation the second lamella was divided, followed by careful dissection of the retrosternal tissue. The heart–lung machine was mounted on the table parallel to the sternal opening in regular cases, whereas in redos all equipment was ready before sternal skin incision including the femoral cannulation setup. Intraoperatively, a cell saver and point of care management for the coagulation system and platelet analysis were used routinely in all cases, while heparin was restarted after cessation of postoperative bleeding.

Our results are in accordance with a previous single-center study analyzing the impact of resternotomy in 100 patients, who received continuous-flow LVAD (HeartMate II, *n* = 93; Heartware, *n* = 7) [13]. In this study, patients in the resternotomy group also exhibited a higher risk profile at baseline, but in contrary to our results, they were significantly longer on CPB. The study showed that postoperative complications were similar for the two groups, except for bleeding requiring re-exploration, which was higher in the resternotomy group, whereas the transfusion rates did not differ significantly. This was attributed by the authors to their policy of taking patients back to the operating room early before significant blood loss occurs and this practice may explain the higher rates of re-exploration. An old report on 135 pulsatile-flow devices implanted as BTT therapy with 53% reported rate of resternotomy demonstrated that resternotomy did not have an impact on the rates of severe bleeding requiring re-exploration, perioperative RVAD support, or survival to transplant [14]. Although referring to the previous generation devices and lacking the long-term experience of the current era, this study implies too that the risk of resternotomy in LVAD surgery may be overestimated. The most extensive research on this topic was conducted in non-LVAD populations. However, these studies have yielded controversial results. Additionally, the heterogeneity of the studied populations precludes a direct comparison to our study [3–11]. An extended review article on the published literature concludes that, due to the discrepancies in the reported rates of complications, a clear temporal trend towards better outcomes in the recent era is not supported by the current evidence [9].

### Follow-up outcome

Concerning the follow-up outcome, the rates of observed complications and the hospitalization outcomes at 6 months post-implantation were not significantly influenced by resternotomy. Patients with first time sternotomy did not exhibit a survival benefit, as demonstrated by the Kaplan–Meier method. Adjusted risk factor analysis revealed that DT was the only strong predictor of death, with nearly a threefold increase in relative risk. The largest study to report on the long-term outcome of LVAD patients to date is the INTERMACS study. This demonstrated that history of cardiac surgery and history of CABG are independent risk factors for death. The IMACS registry, which has practically replaced the INTERMACS, also reported an association of prior CABG with the risk of death in a more contemporary population [2]. However, these reports exhibit substantial differences from our study. Cardiac surgery is not invariably associated with median sternotomy, as a growing number of operations, especially for valve surgery, are conducted through lateral thoracotomy as minimally invasive procedures. As a result, a number of patients included in the INTERMACS study may have had previous cardiac surgery without sternotomy. Furthermore, the INTERMACS included LVADs, as well as biventricular assist devices (BiVADs), and the IMACS registry LVADs, RVADs, BiVADs, and total artificial hearts (TAHs) in the risk factor analysis. Patients receiving BiVAD or TAH had a significantly worse survival than LVAD patients in these studies and this may have led to a risk overestimation. Three different types of continuous-flow LVAD were included in the INTERMACS analysis (Thoratec HeartMate II, HeartWare HVAD, MicroMed DeBakey Child VAD) and several implant sites contributed to data collection. As a result, variations in surgical experience and quality of postoperative care are inevitable. In addition, these studies conducted a longer follow-up. Considering the higher risk profile of the redo group, it is reasonable that associated comorbidities of the reoperated patients will prevail as determinants of outcome, as follow-up extends in time. Our study enrolled a relatively small number of patients receiving HVAD. All patients were operated by the same surgical team and received a standardized postoperative and follow-up care. The HVAD is a small, intrapericardial, centrifugal-flow LVAD that was recently found to be non-inferior to an axial-flow LVAD (HeartMate II) for DT, with respect to survival free from disabling stroke or device removal for malfunction or failure [15]. The small pump size, which allows an entirely intrapericardial placement without the need for pump-pocket preparation, may be advantageous in the context of an altered anatomical field at sternal reentry.

It is of interest that DT emerged as an independent predictor of death in our study, as well as in the aforementioned registries. In spite of that, DT must be cautiously treated as an independent variable with prognostic utility, as it is rather a vague and unspecific concept that often represents a more complex clinical profile of the patient. Advanced heart failure patients are assigned to DT if they are too sick and too old to be considered for heart transplantation and not sick enough to be offered a conservative or palliative therapy. Comorbidities, such as a severe irreversible pulmonary hypertension and older age, preclude bridging therapies, whereas long-term LVAD therapy is considered risky but promising. As such, DT should be rather treated as a surrogate for other risk factors that eventually impact upon adverse outcomes.

### Limitations

Our study has the inherent limitations of a retrospective, non-matched cohort study. The sample size was relatively small. As we studied only one device type, our results may not be extrapolated to other LVADs or durable MCS devices. In addition, the majority of patients were assigned to DT and that is accompanied by an underrepresentation of the bridging therapies. Furthermore, data regarding the perioperative management of redo cases were not available for analysis. It is possible that a longer follow-up would uncover higher death rates, as the resternotomy group exhibits a significantly higher risk profile at baseline. However, our results suggest that with standardized approaches excellent acute results can be reached also in redo patients undergoing LVAD implantation.

### Future outlook

During the recent years, several centers adopted a minimally invasive, sternum-sparing technique for LVAD implantation [16–21]. The authors reported that the reduction in surgical trauma is associated with less blood loss, as well as lower infection and hospitalization rates. However, concomitant cardiac procedures are usually not feasible, due to the limited operative field, while there are only limited data regarding the long-term outcome of these practices. Currently, the gold standard approach for device placement is through a median sternotomy. Taking into consideration the expanding indications for LVAD therapy and the increasing prevalence of heart failure, it is anticipated that the rates of prior sternotomy among LVAD candidates will rise.

## Conclusion

Approximately 20% of patients receiving LVAD have a history of median sternotomy for cardiac surgery. These patients are older and exhibit a higher risk profile at baseline and the vast majority are assigned to DT. The implantation of an intrapericardial continuous-flow LVAD through median redo-sternotomy, although technically challenging, is not associated with higher rates of perioperative complications, longer hospital stay, and worse survival. We advocate that standardized perioperative treatment algorithms, such as close surveillance of the coagulation system, surgical instrumentation, and early postoperative care, have the potential to counterbalance the risks of resternotomy. The focus of ongoing research should be directed to other possible determinants of prognosis, including the optimal time point for device placement and device-associated complications.
